# LCN2 Is a Potential Biomarker for Radioresistance and Recurrence in Nasopharyngeal Carcinoma

**DOI:** 10.3389/fonc.2020.605777

**Published:** 2021-02-02

**Authors:** Meng-Xia Zhang, Li Wang, Lei Zeng, Zi-Wei Tu

**Affiliations:** ^1^State Key Laboratory of Oncology in South China, Department of Nasopharyngeal Carcinoma, Sun Yat-Sen University Cancer Center, Collaborative Innovation Center for Cancer Medicine, Guangzhou, China; ^2^Department of Radiotherapy, Eye & ENT Hospital, Fudan University, Shanghai, China; ^3^Department of Oncology, The Second Affiliated Hospital of Nanchang University, Nanchang, China; ^4^NHC Key Laboratory of Personalized Diagnosis and Treatment of Nasopharyngeal Carcinoma (Jiangxi Cancer Hospital of Nanchang University), Nanchang, China

**Keywords:** nasopharyngeal carcinoma, lipocalin 2, hypoxia-inducible factor 1-alpha, radioresistance, recurrence

## Abstract

**Background:**

Radioresistance-induced local failure, which can result in residual or recurrent tumors, remains one of the major causes of treatment failure in nasopharyngeal carcinoma (NPC). Lipocalin 2 (LCN2) is known to play important roles in cancer initiation, progression, and treatment responses. However, its role in the radioresistance of NPC remains unclear.

**Methods:**

Microarray data from the Gene Expression Omnibus (GEO) was screened for candidate biomarkers relating to the radioresistance of NPC. The expression of LCN2 in NPC cell lines was verified by quantitative real-time PCR (RT-qPCR) and western blotting. The effects of knockdown or overexpression of LCN2 on NPC radiosensitivity were examined using a soft agar colony formation assay and a *γ*H2AX assay. LCN2 expression in NPC specimens was evaluated by immunohistochemistry. Survival outcomes were analyzed. A possible correlation between LCN2 and hypoxia-inducible factor 1-alpha (HIF-1A) was examined by western blotting and a tissue microarray.

**Results:**

LCN2 was highly expressed in the radioresistant NPC cell line CNE2R. Knocking down LCN2 enhanced the radiosensitivity of NPC cells by impairing their ability to repair DNA damage or proliferate, while ectopic expression of LCN2 conferred additional radioresistance to NPC cells. Immunohistochemical analysis of 100 NPC specimens revealed that LCN2 expression was significantly upregulated in radioresistant NPC tissues and was associated with NPC recurrence. Furthermore, a significant correlation between the expression of LCN2 and HIF-1A was detected.

**Conclusion:**

LCN2 is associated with radioresistance and recurrence in NPC and may facilitate the development of a radioresistant phenotype through interacting with HIF-1A. Our data indicate that LCN2 is a promising target for predicting and overcoming radioresistance in NPC.

## Introduction

Nasopharyngeal carcinoma (NPC) is a malignancy of the epithelial origin. Although rare in western countries, it is endemic in Southeast Asia and southern China. NPC age-standardized incidence rates are 3.0 and 0.4 per 100,000 population for China and western countries, respectively (1). NPC is one of the most frequently diagnosed malignancies in China (2). Radiotherapy is the primary treatment option for non-metastatic NPC owing to the high sensitivity of this cancer to ionizing radiation and the relatively inaccessible anatomical location of the nasopharynx, which renders surgery difficult to implement. However, the efficacy of radiotherapy and the prognosis of NPC patients are impaired by inherent or acquired radioresistance, which can result in tumor recurrence or distant failure (3). Consequently, investigating the mechanism of radioresistance and identifying biomarkers that can predict radioresistance and outcomes for NPC patients are urgently needed to allow for individualized treatment.

Lipocalin 2 [LCN2; also known as neutrophil gelatinase-associated lipocalin (NGAL)] is a 25-kDa protein belonging to the lipocalin superfamily and is a vital modulator of iron homeostasis (4). A growing number of studies have identified the *LCN2* gene as crucial for various tumor-related processes, including tumorigenesis, tumor progression, and tumor resistance to therapies such as radiotherapy, chemotherapy, endocrine therapy, and targeted therapy (5–12). A previous study has demonstrated that LCN2 is upregulated in lung cancer cells treated with X-ray irradiation and the sensitivity of these lung cancer cells to radiation is enhanced by the silencing of LCN2. Additionally, LCN2 overexpression has been associated with radioresistance in both oral cancer and lung cancer cells and can serve as a predictor of radioresistance (13). These findings indicated that LCN2 may play an important role in the radioresistance of several tumors. NPC is highly sensitive to radiotherapy and is markedly different from other head and neck cancers (*e.g.*, oral cancer). However, the functional role of LCN2 in NPC radioresistance remains largely unknown.

In the current study, we assessed the potential of *LCN2* as a biomarker for NPC radioresistance through analyzing a Gene Expression Omnibus (GEO) data set (GSE48501). Furthermore, we also investigated the relationship between LCN2 and radioresistance, recurrence of NPC. Our results suggested that LCN2 may be an important biomarker for NPC and throw light on the potential mechanisms underlying NPC radioresistance.

## Materials and Methods

### Cell Lines

NPC cell lines (CNE1, HNE1, HNOE1, SUNE1, CNE-2, and its radioresistant cell type CNE2R) were obtained from Sun Yat-Sen University Cancer Center (Guangzhou, China) and maintained in DMEM medium (Invitrogen, California, USA) supplemented with 10% fetal bovine serum (Gibco, New York, USA) and 1% penicillin–streptomycin (HyClone, Utah, USA). All cells were cultured at 37°C in a humidified chamber with 5% CO_2_.

### Microarray Data Analysis

The mRNA expression profile of gene chip GSE48501 was downloaded from the GEO database (https://www.ncbi.nlm.nih.gov/geo/query/acc.cgi?acc=GSE48501) (14). GSE48501 includes the expression profile of two human radioresistant NPCs and two human radiosensitive NPCs obtained using the Affymetrix Human Genome U133 Plus 2.0 Array platform. Raw data were preprocessed using the Bioconductor package ‘affy’ as previously described (15). Differentially expressed genes (DEGs) were analyzed using the GEO2R tool (http://www.ncbi.nlm.nih.gov/geo/geo2r/). Adjusted P-value <0.01 was used to select DEGs.

### Patients and Tissue Specimens

A total of 100 primary-diagnosed, non-disseminated, paraffin-embedded NPC tissue specimens were obtained from Jiangxi Provincial Hospital of Nanchang University (Nanchang, China) from February 2011 to November 2015 for immunohistochemical analysis. The sensitivity of NPC patients to radiotherapy was defined as previously described (10, 16). In brief, patients with radioresistant NPC were defined as those with incomplete regression of lesions after radical irradiation; residual tumors at more than 6 weeks after the completion of radiotherapy; or local/regional recurrence after radiotherapy. Patients with radiosensitive NPC were defined as those with complete regression after irradiation or without recurrence after the completion of radiotherapy (16). Written informed consent was obtained from all the patients. Approval for NPC tissue use was granted by the Ethics Committee of Jiangxi Provincial Hospital.

### Western Blotting

Western blotting was performed to verify the knockdown or overexpression of LCN2 in NPC cells. Cells were rinsed with cold phosphate-buffered saline (PBS) and lysed in RIPA buffer (Beyotime, Shanghai, China). The lysates were then incubated on ice for 30 min and centrifuged at 12,000 rpm for 25 min at 4°C. Equal amounts (40 µg) of protein were separated by 12% sodium dodecyl sulfate–polyacrylamide gradient gel electrophoresis (SDS–PAGE) and transferred onto polyvinylidene difluoride (PVDF) membranes. The membranes were blocked with 5% skimmed milk for 2 h at room temperature and then incubated with a 1:2,000 dilution of an anti-LCN2 rabbit polyclonal antibody (TA322583, Origene, Maryland, USA) for 16 h at 4°C. This was followed by incubation with a 1:5,000 dilution of a horseradish peroxidase-conjugated secondary antibody for 1 h at room temperature on a shaker. An enhanced chemiluminescence reagent (Thermo Scientific, Massachusetts, USA) was used to detect protein signals. GAPDH was used as a loading control.

### Quantitative Real-Time PCR

Total RNA was extracted from NPC cells using TRIzol reagent (Invitrogen) according to the manufacturer’s instructions. The concentration and quality of the isolated RNA were evaluated using an Agilent 2100 Bioanalyzer (Agilent Technologies, California, USA). First-strand cDNA was reverse-transcribed using the Prime-Script RT Reagent Kit with gDNA Eraser (TaKaRa, Tokyo, Japan). Quantitative real-time PCR (qPCR) was performed to measure *LCN2* mRNA levels using SYBR Premix Ex Taq II (TaKaRa). The sequences of the primers used were 5′-GCTGACTTCGGAACTAAAGGAGAA-3′ (forward) and 5′-GGGAAGACGATGTGGTTTTCA-3′ (reverse) for *LCN2* and 5′-CATCTCTGCCCCCTCTGCTGA-3′ (forward) and 5′-GGATGACCTTGCCCACAGCCT-3′ (reverse) for *GAPDH*, which was used as an internal control (13). The PCR cycling parameters were as follows: 95°C for 30 s, followed by 40 cycles of 95°C for 20 s, 60°C for 30 s, and 70°C for 2 min. All the reactions were performed in triplicate. Gene expression was normalized that of *GAPDH* and quantified using the 2-ΔΔCt method.

### Plasmids, RNA Interference, and Stable Transfection

Human *LCN2* cDNA or a negative control sequence was cloned into a pSin-EF2 retroviral vector (Origene). CNE2R and HNE1 cells stably expressing scrambled or LCN2 short hairpin RNAs (shRNAs) were established by the Sigma shRNA system according to the manufacturer’s instructions. The sequences for human LCN2 shRNA-1 and shRNA-2 were 5′-TACAATGTCACCTCCGTCCTGTTTAGGAA-3′ and 5′-GAGAACCAAGGAGCTGACTTCGGAACTAA-3′, respectively; the non-specific shRNA control sequence was 5′-GCACTACCAGAGCTAACTCAGATAGTACT-3′. The constructed vectors were verified by DNA sequencing and then transfected into 293T cells. The supernatants containing the lentiviruses were collected and purified at 72 h post-transfection. Transfected cells were selected with puromycin (Sigma–Aldrich, St. Louis, MO, USA) at a concentration of 2 μg/ml or neomycin (InvivoGen, Hong Kong, China) at a concentration of 300 μg/ml for 1–2 weeks. To determine transfection efficiency, LCN2 protein levels were assessed by western blotting.

### Soft Agar Colony Formation Assay

The soft agar colony formation assay was performed as previously described (17, 18). Briefly, 100, 200, 1 × 10^3^, or 1 × 10^4^ cells were suspended in 2 ml of 0.6% top agar (Sigma–Aldrich) and plated onto 1.2% base agar in 6-well plates and irradiated with a 0-, 2-, 4-, or a 6-Gy dose of 160 kV X-rays (RAD SOURCE, USA). The irradiated cells were cultured for 14 days. Colonies with a diameter of >50 µm were counted and imaged at ×4 magnification using a Nikon ECLIPSE Ti2 inverted fluorescence microscope. The cloning efficiency was calculated by dividing the number of colonies by the number of cells plated. Each measurement was the average ± standard deviation (SD) of three experiments.

### *γ*H2AX Assay

Cells were plated in 30-mm dishes and cultured for 72 h at 37°C. To detect irradiation-induced DNA double-strand breaks (DSBs), cells were treated with a 2-Gy dose of irradiation from an external X-ray source (RAD SOURCE) at room temperature and incubated for 0.5 and 24 h. Unirradiated cells served as controls. To detect H2AX phosphorylation, cells were sequentially fixed in 4% formaldehyde (Sigma–Aldrich) for 15 min and 50% methanol in PBS for 10 min. The cells were subsequently blocked with 5% bovine serum albumin for 30 min, incubated with a rabbit monoclonal anti-*γ*H2AX antibody (1:1,000, Cell Signaling Technology, Boston, USA) for 30 min, washed in PBS, incubated with an Alexa 488-conjugated (Molecular Probes, USA) secondary antibody for 30 min, and counterstained with DAPI (Invitrogen). Images were captured using an Olympus FV100 confocal microscope. *γ*H2AX-positive cells were defined as those with more than 20 *γ*H2AX foci. Five random fields per coverslip were selected to calculate the number of *γ*H2AX-positive cells. Assays were performed in triplicate to eliminate intra-assay variability.

### Immunohistochemistry

Immunohistochemical analysis of LCN2 was performed on 100 paraffin-embedded NPC specimens. First, tissue slides were baked in an oven at 60°C for 2 h and deparaffinized twice with dimethylbenzene, 10 min each step, and rehydrated with graded ethanol. The slides were then treated with citrate buffer (pH 6.0) under high pressure for antigen retrieval followed by the blocking of endogenous peroxidase activity with 0.3% H_2_O_2_ for 30 min. Next, the slides were sequentially incubated with an anti-LCN2 antibody (1:100 dilution, Origene) for 16 h at 4°C, a biotinylated anti-rabbit antibody (1:1,000 dilution) for 30 min at room temperature, and a biotinylated secondary antibody for 1 h at 37°C. Finally, the tissue sections were stained with 3,3′-diaminobenzidine tetrahydrochloride (DAB) and counterstained with Harris modified hematoxylin. The immunohistochemical results were scored as the intensity grades multiplied by the positive ratios, as previously reported (19). The scores were classified as 0–3 (no staining, weak staining, moderate staining, and strong staining) for the staining intensity and 0–4 (no staining, <10, 10–50, 50–80, and >80% staining) for the positive ratio. The final scores (0–12) were grouped into no/low expression (≤6) and high expression (>6). The scores were determined blindly by two pathologists.

### Statistical Analyses

The data are presented as means ± SD from three independent tests. The Student’s *t*-test or the Mann–Whitney *U* test was used to compare the differences between continuous parameters. The distribution of clinicopathological variables between high and low LCN2 expression groups was compared by the chi-square test. Survival curves were constructed using the Kaplan–Meier method and compared using the log-rank test. The endpoints were assessed as follows: local relapse-free survival (LRFS) and nodal relapse-free survival (NRFS) were measured from the date of treatment to the date of the first observation of local and regional recurrence, respectively. Local and regional relapse were defined as relapse-free survival (RFS). Distant metastasis-free survival (DMFS) was measured from the date of treatment to the date of the first observation of distant metastasis. Progression-free survival (PFS) was measured from the date of treatment to either the date of the first observation of local or regional recurrence, or distant metastasis. Overall survival (OS) was measured from the first date of treatment to the date of death due to any cause. Multivariable analysis was conducted using the Cox proportional hazards model after adjusting for confounding factors such as age, sex, T stage, N stage, and receiving or not induction chemotherapy. The significance of any correlation between LCN2 and HIF-1A expression was determined by Pearson’s correlation analysis. All statistical tests were two-sided. Associations were considered statistically significant at *P*-values <0.05. All statistical analyses were performed using SPSS version 26.0 (SPSS IBM, Chicago, USA). The raw data obtained in this study have been uploaded onto the Research Data Deposit (RDD) with the RDD number RDDB2020000932.

## Results

### LCN2 Was Identified as a Radioresistance-Related Gene in NPC

**Figure 1:**
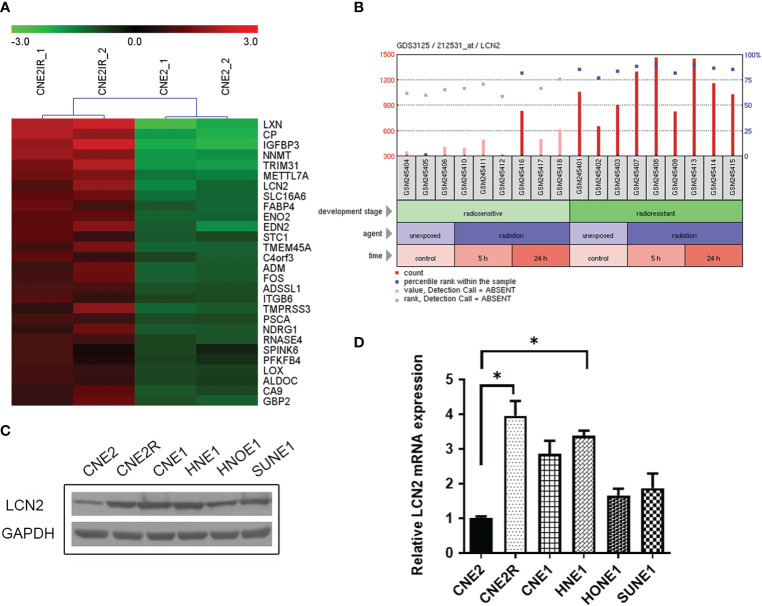
LCN2 was identified as a radioresistance-related gene in nasopharyngeal carcinoma (NPC). **(A)** A heatmap of the overlapping differential expressed genes (DEGs) between the radiosensitive cell line CNE2 and the radioresistant cell line CNE2R in GSE48501. **(B)** The responses of radiosensitive and radioresistant squamous cell carcinomas (SCCs) to ionizing radiation (time course). LCN2 expression was upregulated in both radioresistant and radiosensitive SCCs after irradiation. **(C, D)** Validation of LCN2 expression in NPC cell lines. LCN2 protein **(C)** and mRNA **(D)** levels were examined by western blotting and quantitative real-time PCR (RT-qPCR), respectively. GAPDH was used as an internal control. *P < 0.05.

We manually found a GEO data set (GSE48501), using which, we conducted a comparative analysis of mRNA expression in NPC cell lines CNE2 and CNE2R. The online analysis tool GEO2R showed the upregulation of LCN2 in CNE2R compared to that of CNE2 (**Figure 1A**). A GEO profile was found at GDS3125/212531_at (nih.gov) and demonstrated the responses of radiosensitive and radioresistant tumors to ionizing radiation (time course). Briefly, squamous cell carcinoma-derived xenografts were generated and allowed to grow to a volume of 150–200 mm^3^. At that time, tumors were either treated with a 3-Gy dose of irradiation or left untreated and then collected for RNA purification 5 or 24 h later. The overall expression level of LCN2 was higher in the radioresistant squamous cell carcinomas (SCCs) than in the radiosensitive SCCs, in both the irradiated and untreated groups (*P* < 0.0001). In the radiosensitive SCCs, LCN2 expression was significantly upregulated at the 24-h time point after irradiation when compared with that in the untreated group (*P* = 0.0441) **(****Figure 1B** and **Supplementary Table 1**).

### Validation of LCN2 Expression in NPC Cell Lines

To verify the expression of LCN2 identified in the microarray data, western blotting and RT-qPCR were performed to detect the protein and mRNA levels of LCN2, respectively, in five NPC cell lines (CNE1, CNE2, HNE1, HNOE1, and SUNE1) and one radioresistant NPC cell line (CNE2R). Consistent with the results of the microarray analysis, the highest expression of LCN2 was found in CNE2R cells. Furthermore, LCN2 was also highly expressed in CNE1 and HNE1 cells (**Figures 1C, D**). CNE1 is a highly differentiated NPC-derived squamous cell carcinoma cell line, while HNE1 is an Epstein–Barr virus (EBV)-positive cell line derived from a poorly differentiated squamous carcinoma. According to the law of Bergonié and Tribondeau, highly differentiated tumor cells usually display medium to low sensitivity to radiation (20, 21). In NPC, EBV infection is one of the most important factors contributing to radioresistance (22, 23). Consequently, these results indicate that LCN2 expression is upregulated in radioresistant NPC cells.

### LCN2 Regulates the Radiosensitivity of NPC Cells

**Figure 2:**
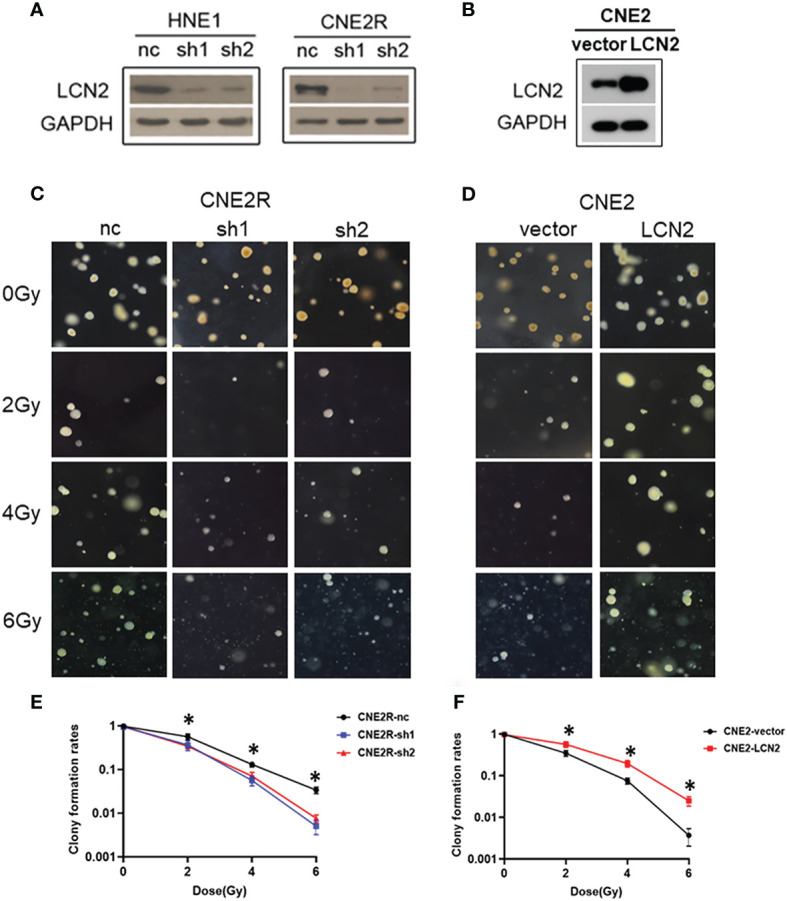
LCN2 regulates the radiosensitivity of nasopharyngeal carcinoma (NPC) cells. **(A, B)** Validation of LCN2 expression in various NPC cell lines. Knockdown or ectopic expression of LCN2 in HNE1, CNE2R, or CNE2 cells was validated by western blotting. **(C, E)** A soft agar colony formation assay was used to assess the radiosensitivity of NPC cells. The colony formation rates were markedly reduced in CNE2R cells with LCN2 knockdown compared with that of control cells following irradiation at the dose of 2, 4, or 6 Gy. *P < 0.05. **(D, F)** CNE2 cells overexpressing LCN2 formed more colonies than control cells at the irradiation doses of 2, 4, and 6 Gy. *P < 0.05.

To determine whether LCN2 levels contribute to NPC radiosensitivity, we generated stable LCN2-knockdown CNE2R and HNE1 cell lines as well as a CNE2 cell line stably overexpressing LCN2. Stable transfections were confirmed by western blotting (**Figures 2A, B**). The colony survival assay is the gold standard method for assessing the radiosensitivity of tumor cells (24). Here, we performed a soft agar colony assay to investigate the effect of LCN2 on cell proliferation after irradiation. We found that, following irradiation at the dose of 2, 4, or 6 Gy, colony-formation rates were markedly reduced in LCN2-depleted CNE2R cells compared with that of control cells (**Figures 2C, E**). Conversely, LCN2-overexpressing cells formed more colonies than control cells (**Figures 2D, F**).

**Figure 3:**
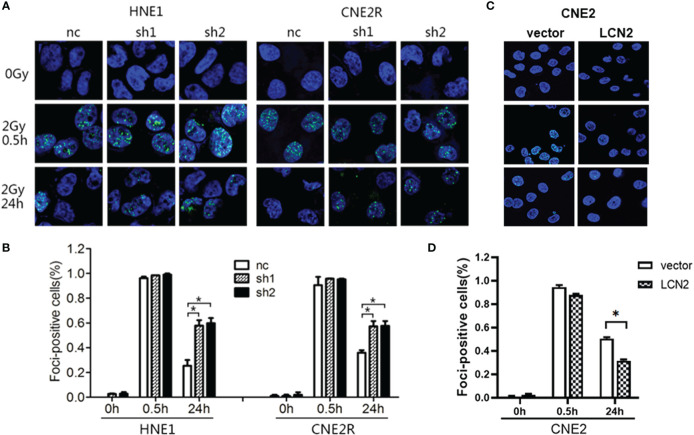
LCN2 regulates the radiosensitivity of nasopharyngeal carcinoma (NPC) cells. **(A, B)** The DNA double-strand break repair capacity was impaired in HNE1 and CNE2R cells with LCN2 knockdown, as evidenced by the greater number of *γ*H2AX-positive LCN2-depleted cells when compared with that of control cells. *P < 0.05. **(C, D)** The DNA double-strand break repair capacity was enhanced in LCN2-overexpressing CNE2 cells based on the percentage of *γ*H2AX-positive cells. *P < 0.05.

Double-strand breaks (DSBs) constitute the major type of DNA damage caused by irradiation (25), while the DSB repair capacity is closely associated with radiosensitivity (26). Therefore, we conducted a *γ*H2AX [a biomarker of DSBs (27)] assay to examine the phosphorylation status of H2AX in these cells at 0, 0.5, and 24 h post-irradiation. At 24 h after receiving a 2-Gy dose of radiation, the DNA damage repair ability of cells with LCN2 knockdown was significantly attenuated (**Figures 3A, B**), whereas the opposite effect was observed in CNE2 cells stably overexpressing LCN2 (**Figures 3C, D**). These results indicated that LCN2 may regulate NPC radiosensitivity by influencing the DNA damage repair process.

### LCN2 Is a Potential Biomarker for Predicting NPC Radioresistance

**Figure 4:**
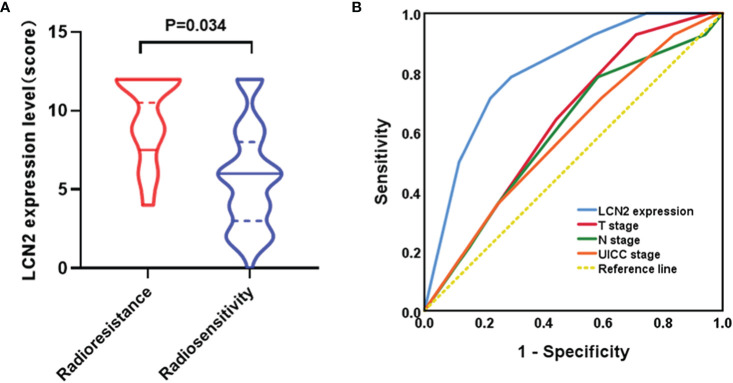
LCN2 is a potential biomarker for predicting nasopharyngeal carcinoma (NPC) radioresistance. **(A)** LCN2 expression was significantly higher in radioresistant NPC tissues than in radiosensitive NPC tissues (*P* = 0.034). **(B)** Receiver operating characteristic (ROC) curves for the predictive value of using LCN2 expression, T stage, N stage, and UICC stage to discriminate between radioresistant NPC and radiosensitive NPC. The LCN2 level displayed the largest area under the curve (AUC) among the four parameters.

A total of 14 patients were defined as having radioresistant NPC according to the definition mentioned in the section *Patients and Tissue Specimens*. Immunohistochemical analysis showed that LCN2 expression was significantly higher in radioresistant NPC tissues than in radiosensitive NPC tissues (*P* = 0.034) (**Figure 4A**). The response to radiotherapy is related to the intrinsic characteristics of NPC, including tumor size and infiltration status. Therefore, we compared the potential of using LCN2 expression with that of using T stage, N stage, or UICC stage to discriminate between patients with radioresistant NPC and those with radiosensitive NPC using receiver operating characteristic (ROC) curves. The area under the curve (AUC) values for LCN2 expression, T stage, N stage, and UICC stage were 0.808, 0.634, 0.600, and 0.588, respectively. The sensitivity and specificity of LCN2, T stage, N stage, and UICC stage were 78.6 and 70.9, 64.3% and 55.8, 78.6 and 41.9%, and 71.4 and 40.7%, respectively (**Figure 4B**). These results indicated that the LCN2 expression level was the best predictor of NPC radioresistance among the four indicators.

### The Association Between LCN2 Expression and Survival Outcomes in NPC

**Figure 5:**
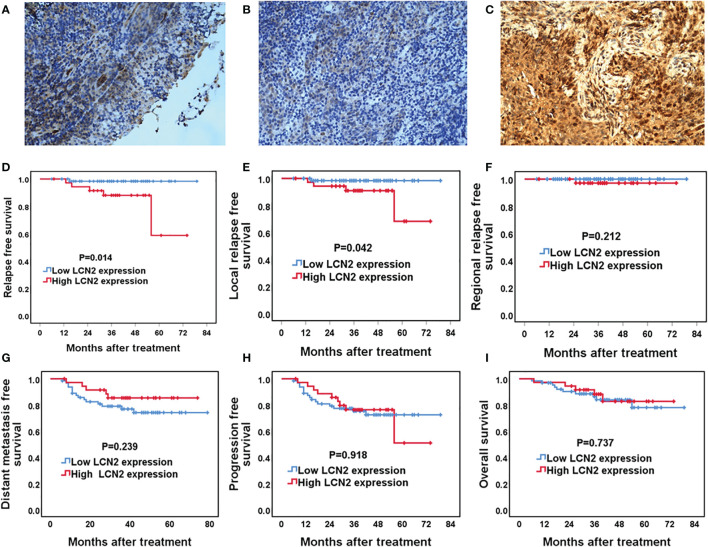
The association between LCN2 expression and survival outcomes in nasopharyngeal carcinoma (NPC) patients. **(A–C)** Representative micrographs (×400) of normal nasopharynx mucosa **(A)**, NPC tissues with low LCN2 expression **(B)**, and NPC tissues with high LCN2 expression **(C)** are shown. **(D–I)** Kaplan–Meier analysis of the 5-year relapse-free survival (RFS), local relapse-free survival (LRFS), regional relapse-free survival (RRFS), distant metastasis-free survival (DMFS), progression-free survival (PFS), and overall survival (OS) between high and low LCN2 expression groups. The RFS and LRFS of patients with high LCN2 expression were significantly lower than those of patients with low LCN2 expression. There were no significant differences in RRFS, DMFS, PFS, or OS between the low and high LCN2 expression groups.

Next, we assessed whether there was a correlation between LCN2 expression and the clinical parameters of 100 NPC patients. Sections of normal nasopharynx mucosa and NPC tissues stained for LCN2 are shown in **Figures 5A–C**. The expression of LCN2 was not significantly associated with age, sex, T stage, N stage, UICC stage, or whether or not patients had received induction chemotherapy (**Table 1**). High expression of LCN2 was associated with poor LRFS (*P* = 0.042) and RFS (*P* = 0.014), but not with NRFS (*P* = 0.212), DMFS (*P* = 0.239), PFS (*P* = 0.918), or OS (*P* = 0.737) (**Figures 5D–I**). Further multivariate analyses identified LCN2 as an independent and unfavorable prognostic indicator for RFS in NPC patients (*P* = 0.022) (**Table 2**). We also found that LCN2 expression was an independent prognostic factor for LRFS, but with borderline significance (*P* = 0.055). Radioresistance is known to facilitate tumor recurrence to some extent. Consequently, these results indicated that LCN2 overexpression is clinically relevant for NPC recurrence.

**Table 1:** The associations between LCN2 expression and clinicopathological parameters.

Variable	Total population	LCN2 expression level		*P*-value
		Low	High	
**Sex**				0.738
Female	34	21	13	
Male	66	43	23	
**Age** (y)				0.129
<50	51	29	22	
≥50	49	35	14	
**T stage**				0.089
T1–3	38	15	53	
T4	26	21	47	
**N stage**				
N0–1	26	13	39	0.657
N2–3	38	23	61	
**UICC stage**				
I–III	28	11	39	0.194
IV	36	25	61	
**Induction CT**				
No	23	9	32	0.260
Yes	41	27	68	

CT, chemotherapy.

**Table 2:** Multivariate analysis using a Cox proportional hazards model for the RFS and LRFS of NPC patients.

Endpoint	Variables	HR	95% CI	P-value
RFS	Sex (female *vs.* male)	0.990	[0.172–5.697]	0.991
	Age (<50 *vs.* ≥50)	2.212	[0.401–12.215]	0.362
	T stage (T1–3 *vs.* T4)	0.401	[0.068–2.376]	0.314
	N stage (N0–1 *vs.* N2–3)	0.595	[0.097–3.644]	0.575
	Induction CT (no *vs.* yes)	0.823	[0.133–5.085]	0.834
	**LCN2 expression (low *vs.* high)**	13.925	[1.360–142.548]	**0.026**
LRFS	Sex (female *vs.* male) Age (<50 *vs.* ≥50) T stage (T1–3 *vs.* T4) N stage (N0–1 *vs.* N2–3) Induction CT (no *vs.* yes) **LCN2 expression (low *vs.* high)**	0.515 4.484 0.151 1.179 1.870 12.999	[0.070–3.816] [0.574–35.048] [0.015–1.484] [0.152–9.150] [0.152–22.952] [0.950–177.791]	0.516 0.153 0.105 0.875 0.625 **0.055**

RFS, relapse-free survival; LRFS, local relapse-free survival; HR, hazard ratio; CI, confidence interval; CT, chemotherapy. The bold values highlight meaningful p-values with statistical significance or borderline significance.

### The Correlation Between the Expression of LCN2 and HIF-1A

**Figure 6:**
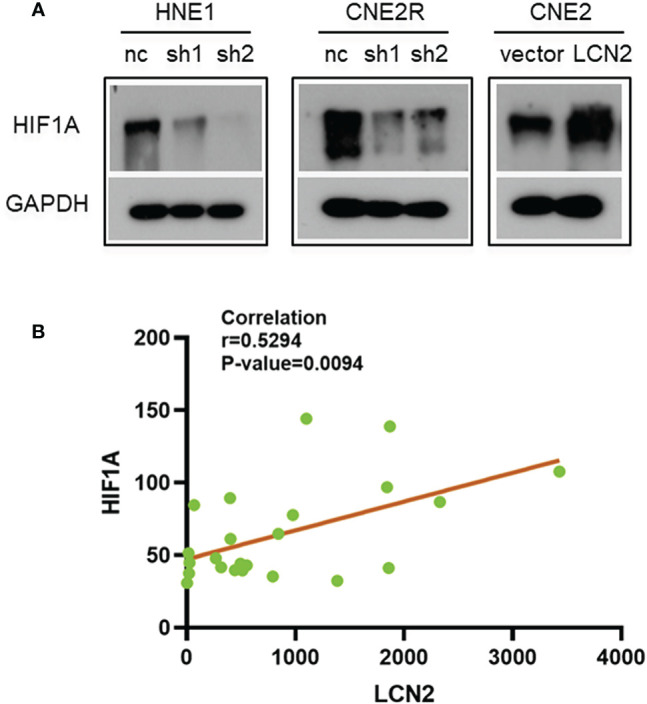
The correlation between the expression of LCN2 and that of hypoxia-inducible factor 1-alpha (HIF-1A). **(A)** The protein level of the hypoxia-related factor HIF-1A was reduced in both HNE1 and CNE2R cells with LCN2 knockdown, but was increased in CNE2 cells stably overexpressing LCN2. **(B)** A significant correlation was detected between LCN2 and HIF-1A expression in 23 NPC tissues using a microarray (Pearson correlation coefficient (*r*) = 0.5294, *P* = 0.0094).

Given that radioresistance is closely related to the hypoxic microenvironment of tumors (28, 29), we speculated that there may be a correlation between the expression of LCN2 and that of hypoxia-related genes. We found that the protein level of HIF-1A, a hypoxia-related factor, was reduced in both HNE1 and CNE2R cells with LCN2 knockdown, while it was increased in CNE2 cells stably overexpressing LCN2 (**Figure 6A**). We also performed a correlation analysis between LCN2 and HIF-1A expression in 23 NPC tissues using a microarray. The results demonstrated that the expression of LCN2 in NPC tissues was positively correlated with that of HIF-1A (Pearson correlation coefficient (*r*) = 0.5294, *P* = 0.0094) (**Figure 6B**). These results suggested that LCN2 may regulate NPC radioresistance through interacting with HIF1A.

## Discussion

Although several genes, including those associated with cell-cycle control, DNA damage repair, and apoptosis are known to influence the effects of ionizing radiation-induced cell damage, our knowledge of radiation-induced resistance in tumors at the molecular level remains limited. Microarrays have been applied to identify genes involved in the radioresistance of various tumors (30–35). Chang et al. analyzed the gene expression profiles of radioresistant NPC cell lines using a cDNA array and identified at least two genes, *GP96* and *GDF15*, that were involved in the development of radioresistance in NPC (35). In this study, we further identified LCN2 as a radioresistance-related gene in NPC cells using the GEO data set GSE48501 and the online analysis tool GEO2R. Additional functional studies and survival analysis confirmed the key role of LCN2 in the acquisition of a radioresistant phenotype and the recurrence of NPC.

Several studies have reported that the aberrant expression of LCN2 can confer resistance to radiotherapy and chemotherapy in several types of cancer (5–13). Additionally, although increased LCN2 expression was shown to correlate with the apoptosis induced by several reagents in human lung cancer cells, this LCN2 upregulation represented a survival rather than a proapoptotic response (36). Meanwhile, LCN2 was also upregulated in HepG2 cells following irradiation or H_2_O_2_ treatment (37). The results of these studies suggest that LCN2 protects tumor cells against extracellular stimuli-induced damage, thereby facilitating their survival. Irradiation-induced cell death results from irreparable DNA DSBs, while radiosensitivity is tightly linked to the ability of cells to repair DNA damage after irradiation (38, 39). DSB repair usually begins within 30–60 min of irradiation and peaks after 24 h. *γ*H2AX is a marker for DSB recognition and repair, and the DSB repair efficacy is characterized by the presence of *γ*H2AX foci (40–44). In this study, we found that knocking down LCN2 markedly impaired the DNA DSB repair capability of the NPC cell lines CNE2R and HNE1 and reduced their proliferative ability, which enhanced the sensitivity of these cells to irradiation. Conversely, the overexpression of LCN2 increased the radioresistance of NPC cells. These results suggest that LCN2 may induce radioresistance *via* regulating the DNA DSB repair capability of NPC cells. Cancer cells can activate several pathways to repair DSBs and maintain their proliferation status, thereby promoting tumor radioresistance and recurrence.

Radioresistance frequently underlies tumor recurrence. In line with this phenomenon, our results showed that patients with high LCN2 expression levels had shorter RFS and LRFS. However, studies investigating LCN2 in different head and neck cancers have reported inconsistent results. LCN2 expression was reported to be downregulated in oral cancer, and was further reduced in oral cancer with metastasis (5, 12, 45). In these studies, patients with high levels of LCN2 had better survival outcomes, making LCN2 a good prognostic factor in oral cancer. The mechanism through which LCN2 exerts its anti-tumor effects in oral cancer may be related to a reduction in autophagy mediated through mTOR signaling pathway activation (12). In contrast, LCN2 was reported to be highly expressed in thyroid carcinoma (46, 47) and the silencing of LCN2 attenuated cancer cell survival under conditions of serum deprivation. The discrepancies among these results are partially due to the high heterogeneity among head and neck cancers. One study demonstrated that survival outcomes for NPC patients with metastasis are generally poor as the biology of NPC differs from that of classic head and neck squamous cell carcinoma (48). NPC tends to be more sensitive to ionizing radiation than other head and neck cancers. Therefore, once NPC becomes resistant to radiotherapy, the treatment outcomes can be poor.

Solid tumors usually have inefficient vasculatures and high energy requirements, resulting in oxygen deprivation (hypoxia) in the tumor microenvironment. Cancer cells can be radioresistant under hypoxic conditions (49, 50). Sørensen et al. reported that, although head and neck cancer cells with HPV infection exhibited markedly greater radiosensitivity than HPV-negative cells, both cell types displayed the same radioresistance potential under hypoxic conditions (51). HIF-1A, which mediates adaptive responses to hypoxia, has been implicated in the induction of biological radioresistance in cancer cells under oxygen deprivation. Most tumor hypoxia adaptations are orchestrated by HIF-1A (52–54). We found that the protein level of HIF-1A was reduced in both HNE1 and CNE2R cells with LCN2 knockdown, but was increased in CNE2 cells stably overexpressing LCN2. Furthermore, we also identified a positive correlation between LCN2 and HIF-1A expression in 23 NPC tissues. Similarly, Yang and et al. demonstrated that LCN2 significantly enhanced VEGF-induced angiogenesis in human breast cancer and that this effect was mediated through HIF-1A *via* extracellular signal-regulated kinase (Erk) (55). LCN2 expression was also reported to be increased in tumor cells cultured under hypoxic conditions and paralleled the levels of HIF-1A in mouse melanoma cells (56). As HIF-1A is firmly associated with the radioresistance of cancer cells, we think that LCN2 might interact with HIF-1A to facilitate the development of a radioresistant phenotype in NPC. Further studies are warranted to elucidate the mechanism underlying how LCN2 and HIF-1A regulate NPC radioresistance.

This study had several limitations. First, the patient population was relatively small, and these results need to be further verified in a larger cohort. Additionally, no *in vivo* experiments were performed. Finally, we did not further explore the mechanism through which LCN2 exerts its effect on the radioresistance of NPC.

In summary, we demonstrated that LCN2 was positively correlated with the radioresistance of NPC cells. LCN2 was highly expressed in patients with radioresistant NPC. Survival analysis revealed that high LCN2 expression was related to poor RFS and LRFS. Additionally, we identified a positive correlation between LCN2 and HIF-1A expression, which suggested that LCN2 may induce NPC radioresistance through regulating pathways associated with adaptation to hypoxia in the tumor microenvironment. The underlying molecular mechanisms remain to be further elucidated.

## Data Availability Statement

The original contributions presented in the study are included in the article/**Supplementary Material**. Further inquiries can be directed to the corresponding authors.

## Ethics Statement

The studies involving human participants were reviewed and approved by the ethics committee of Jiangxi Provincial Hospital. The patients/participants provided their written informed consent to participate in this study.

## Author Contributions

Z-WT and LZ conceived the study. M-XZ and LW helped to organize and perform the study. Statistical analysis was undertaken by Z-WT. All authors read and approved the final version of the manuscript. M-XZ wrote the manuscript.

## Funding

This project was supported financially by grants from the Natural Science Foundation of China (81802915, 81660452, and 81660453).

## Conflict of Interest

The authors declare that the research was conducted in the absence of any commercial or financial relationships that could be construed as a potential conflict of interest.
