# Corrigendum: LCN2 Is a Potential Biomarker for Radioresistance and Recurrence in Nasopharyngeal Carcinoma

**DOI:** 10.3389/fonc.2021.670714

**Published:** 2021-03-18

**Authors:** Meng-Xia Zhang, Li Wang, Lei Zeng, Zi-Wei Tu

**Affiliations:** ^1^State Key Laboratory of Oncology in South China, Department of Nasopharyngeal Carcinoma, Sun Yat-Sen University Cancer Center, Collaborative Innovation Center for Cancer Medicine, Guangzhou, China; ^2^Department of Radiotherapy, Eye & ENT Hospital, Fudan University, Shanghai, China; ^3^Department of Oncology, The Second Affiliated Hospital of Nanchang University, Nanchang, China; ^4^NHC Key Laboratory of Personalized Diagnosis and Treatment of Nasopharyngeal Carcinoma (Jiangxi Cancer Hospital of Nanchang University), Nanchang, China

**Keywords:** nasopharyngeal carcinoma, lipocalin 2, hypoxia-inducible factor 1-alpha, radioresistance, recurrence

An author name was incorrectly spelled as Lei Zheng. The correct spelling is Lei Zeng.

The authors apologize for this error and state that this does not change the scientific conclusions of the article in any way. The original article has been updated.

